# Time of day for COVID vaccine administration linked to clinical effectiveness

**DOI:** 10.1172/JCI168233

**Published:** 2023-06-01

**Authors:** Laura J. Rasmussen-Torvik

In this issue of the *JCI*, Hazan et al. (1) report on whether time of day of COVID vaccination associates with subsequent breakthrough COVID-19 infection in 1,515,754 patients, using data from a large Israeli health maintenance organization. The authors found evidence to support that the timing of vaccine dosing associated with clinical outcomes. When considering the timing of the second dose in the original two-dose COVID vaccine series, individuals who were vaccinated in the morning (0800–1159 hours) had lower rates of breakthrough infections, emergency room visits for COVID, and hospitalizations for COVID when compared with individuals vaccinated in the evening (1600–1959 hours). However, only the association with breakthrough infections rose to the level of statistical significance. While the magnitude of most observed protective hazard ratios for clinical outcomes when comparing morning with evening administration of doses two and three was modest (hazard ratio range, 0.90–0.99), a larger and statistically significant (*P* = 0.038) protective hazard ratio (hazard ratio = 0.64) was observed for hospitalization when comparing morning administration of second booster shots (dose four) with evening administration. During the study period, only those over the age of 60 years and/or those with a history of immunosuppression were eligible for second boosters. When time of dosing was examined in smaller intervals, the lowest rates of breakthrough infections were found in those vaccinated in the late morning to early afternoon. Interestingly, prior studies looking at time of day of COVID vaccination and subsequent antibody titers have been equivocal (2, 3), and this is the first study to our knowledge that included clinical protection as an outcome (1).

Hazan et al. add to a list of critically important COVID-19 papers using data from the Israeli health care system (1, 4, 5). The rapid adoption and administration of the COVID vaccine (almost entirely BNT162b2) in Israel, coupled with Israel’s integrated payer–provider health care organizations offering mandatory health care coverage and comprehensive electronic health record systems, has provided well-powered, critical data for rapid COVID-19 investigation and reporting. The robust public health infrastructure in Israel also advanced COVID-19 studies. Specifically, the Israeli Ministry of Health required a documented positive SARS-CoV-2 test prior to quarantine and a negative test after quarantine before issuing a certificate of recovery to patients throughout the study period, which meant there was strong incentive to obtain a laboratory diagnosis, even if initial testing occurred at home. Of course, these data sets do have limitations. After running adjusted models and performing sensitivity analyses to explore the effect of measured confounders, the authors noted the possibility that their observations resulted from unmeasured confounding in the data set; they also acknowledged that subsequent investigation should assess additional biomarkers, which might help to determine mechanisms driving this observed association.

The potential explanations for the association between receiving COVID vaccination at certain times of day and clinical protection are not well understood and likely multifactorial in nature, arising from biological, environmental, and social influences. Whether this association holds for other vaccinations or in other populations requires more study, but the findings highlight the importance of biological rhythms in response to interventions. The molecular underpinnings of chronobiological responses to vaccines or medications need further study, as they are relevant to both individual and public health.

## Version 1 06/01/2023

Electronic publication
